# Must They Be Driven to a Last Refuge?

**Published:** 1919-11-15

**Authors:** 


### NURSES AND TRADE UNIONS.—I.

### Must They be Driven to a Last Refuge?

CAN we have a fair-and-square uncensored talk on the thorny subject of nurses and trade unions? It is in everybody's mind, and I think it is a fool's game for those who are interested, for or against, to ignore that which is in our midst. Human nature has many peculiarities, one of which is to ignore the obvious. We sometimes laugh at the ostrich, and while we laugh we are digging our own heads into the sand. I ventured recently to mention trade unionism to a highly intelligent and capable matron. Her first impulse was to look cautiously round to make sure that the door was shut. The mere sound of the word was to this worthy lady a shock, and I felt like a kind of modern Guy Fawkes, carrying gunpowder to blow up a time-honoured edifice. This is neither peculiar nor unique. It is a part of our make-up. There are men with perfectly bald heads who regard with something akin to horror any reference to baldness. Their acute sensitiveness becomes an obsession. In actual fact, this super-sensitiveness only serves to draw attention to something which otherwise would pass unnoticed. People die of cancer, but for some inscrutable reason the word is never mentioned. But everybody knows and passes it along in a whisper. The circulation department becomes really busy when there is a secret. I have often thought that if commercial men had the wit they would, in advertising, appeal more successfully to the public if they wrapped their wares in mystery instead of parading them. I contemplate some day making a fortune by initiating a new system of advertising. In this degenerate age it is the man with a secret who captures public attention.

### NOT A SECRET MYSTERY.

I am not out for making any secret a mystery of trade unionism. I want to talk about it and to get others to do likewise. And let me begin by stating that I do not regard it as a disease. There is nothing malignant about such a union. Trade unions are perfectly legitimate institutions, protected by many Acts of Parliament. It is not I nor you, but the British people, through their election representatives, who have made trade unions exactly what they are. Doctors and lawyers, nurses, artisans, clerks, agricultural labourers, can form trade unions, if it so pleases them, and they can, within the law, strike and, without loss of dignity, carry on what is known as peaceful picketing. It may be very embarrassing, very objectionable, even very disgusting. The fact remains, however, that a trade union is a British institution, established by law, and assented to by King, Lords, and Commons.

May I go a little further even than this. In its own house, and within recent months, the Government has set up in State departments what are known as Whitley Councils, and no Civil servant can take an active part in these Councils unless he is a member of a union. It used to be regarded as heterodox and revolutionary to belong to a union. Now it is heterodox and revolutionary to remain outside. The State invites its employers who belong to unions to devote their brains and their energies to two objects, first, the more effectual discharge of the duties of the department to which they belong, and secondly, to their own betterment. If anybody sees a better way of accomplishing something than the method now adopted, he is invited to articulate. The staff has its chosen representatives, and the department has its official representatives, and it is mandatory that these shall meet not less than four times a year to discuss the well-being of the department and the welfare of the staff. I mention this to demonstrate that unions are not only fashionable, but they have State sanction and approval.

### ONE OBJECT ONLY.

The Nurses Trade Union found its origin in Ireland. It had, and has, one only object—to improve the nurse's conditions of service. The Irish nurse is, without exception, the most poverty-stricken, sweated, ill-used woman in his Majesty's realms. At public meetings she is flattered to death. But when the meetings close down she is relegated to her kennel. Miserable wages and impossible hours of labour—these are the only rewards that she actually handles. The much-boosted I.N.A. and I.N.B. touch every subject on earth save one, and that one is the working woman's wages and hours of toil. "Hush! not a word! Registration! Yes. Let us gather round Registration. Let us gather round Representation. We must be on this board and on that. English interference! Thank God for that! We shall gather at our parlours and drink tea to the destruction of the Saxon. The College of Nursing! Here is a new stunt. Let us make war and let us make merry!" Anything and everything, except one thing, and that one thing is the nurse's pay and conditions of service.

I have preached many sermons in this journal on the Economics of Nursing. But they have fallen on deaf ears. For some inscrutable reason there has been, and is, a point-blank refusal to tackle the nurse's economic problem. I make no exception. Mrs. Fenwick has danced on her Registration stunt for thirty years. She made it as fashionable as the jazz. The College of Nursing was lured into competition, and now the Minister of Health is expected to take a turn.

Shadows, ladies and gentlemen all, shadows! There is no money in registration. A Registration Act will accomplish as much and no more for nurses than Lord Knutsford's Charter of Liberty did for probationers. It is like applying sticking-plaster to a gumboil.

*No nurse in England, Scotland, Wales, or Ireland has the slightest desire to belong to a trade union.* Be assured of this fundamental fact. The nurse is not being lured into trade unionism. She is being driven to it as a last refuge. She has paid her subscriptions to many societies, but none has so far lifted a finger to minister to her pressing necessities.

